# Strengthening the reporting of genetic risk prediction studies: the GRIPS statement

**DOI:** 10.1186/gm230

**Published:** 2011-03-15

**Authors:** A Cecile JW Janssens, John PA Ioannidis, Cornelia M van Duijn, Julian Little, Muin J Khoury

**Affiliations:** 1 Department of Epidemiology, Erasmus University Medical Center, PO Box 2040, Rotterdam 3000 CA, The Netherlands; 2 Department of Hygiene and Epidemiology, University of Ioannina School of Medicine, University Campus, Ioannina 45110, Greece; 3 Biomedical Research Institute, Foundation for Research and Technology-Hellas, University Campus, Ioannina 45110, Greece; 4 Department of Medicine, Tufts University School of Medicine, 750 Washington St, Boston, MA 02111, USA; 5 Center for Genetic Epidemiology and Modeling and Tufts CTSI, Institute for Clinical Research and Health Policy Studies, Tufts Medical Center, 750 Washington St, Boston, MA 02111, USA; 6 Stanford Prevention Research Center, Stanford University School of Medicine, 251 Campus Drive, Stanford, CA 94305, USA; 7 Department of Epidemiology and Community Medicine, University of Ottawa, 451 Smyth Rd, Ottawa, Ontario K1H 8M5, Canada; 8 Office of Public Health Genomics, Centers for Disease Control and Prevention, 1600 Clifton Rd, Atlanta, GA 30333, USA

## Abstract

The rapid and continuing progress in gene discovery for complex diseases is fueling interest in the potential application of genetic risk models for clinical and public health practice. The number of studies assessing the predictive ability is steadily increasing, but the quality and completeness of reporting varies. A multidisciplinary workshop sponsored by the Human Genome Epidemiology Network developed a checklist of 25 items recommended for strengthening the reporting of genetic risk prediction studies (the GRIPS statement), building on the principles established by prior reporting guidelines. These recommendations aim to enhance the transparency of study reporting, and thereby to improve the synthesis and application of information from multiple studies that might differ in design, conduct, or analysis. A detailed Explanation and Elaboration document is published at http://www.plosmedicine.org.

## Introduction

The recent successes of genome-wide association studies and the promises of whole genome sequencing fuel interest in the translation of this new wave of basic genetic knowledge to health care practice. Knowledge about genetic risk factors may be used to target diagnostic, preventive, and therapeutic interventions for complex disorders based on a person's genetic risk, or to complement existing risk models based on classical non-genetic factors, such as the Framingham risk score for cardiovascular disease. Implementation of genetic risk prediction in health care requires a series of studies that encompass all phases of translational research [1,2], starting with a comprehensive evaluation of genetic risk prediction.

With increasing numbers of discovered genetic markers that can be used in future genetic risk prediction studies, it is crucial to enhance the quality of the reporting of these studies, since valid interpretation could be compromised by the lack of reporting of key information. Information that is often missing includes details in the description of how the study was designed and conducted (for example, how genetic variants were selected and coded, how risk models or genetic risk scores were constructed, and how risk categories were chosen), or how the results should be interpreted. An appropriate assessment of the study's strengths and weaknesses is not possible without this information. There is ample evidence that prediction research often suffers from poor design and bias, and these may also have an impact on the results of the studies and on models of disease outcomes based on these studies [3-5]. Although most prognostic studies published to date claim significant results [6,7], very few translate to clinically useful applications. Just as for observational epidemiological studies [8], poor reporting complicates the use of the specific study for research, clinical, or public health purposes and hampers the synthesis of evidence across studies.

Reporting guidelines have been published for various research designs [9], and these contain many items that are also relevant to genetic risk prediction studies. In particular, the guidelines for genetic association studies (STrenghtening the REporting of Genetic Association studies - STREGA) have relevant items on the assessment of genetic variants, and the guidelines for observational studies (Strengthening the Reporting of OBservational studies in Epidemiology - STROBE) have relevant items about the reporting of study design. The guidelines for diagnostic studies (STAndards for Reporting Diagnostic accuracy - STARD) and those for tumor marker prognostic studies (Reporting of tumor MARKer studies - REMARK) include relevant items about test evaluation; the REMARK guidelines also have relevant items about risk prediction [10-13]. However, none of these guidelines are fully suited to genetic risk prediction studies, an emerging field of investigation with specific methodological issues that need to be addressed, such as the handling of large numbers of genetic variants (from tens to tens of thousands) and flexibility in handling such large numbers in analyses. We organized a two-day workshop with an international group of risk prediction researchers, epidemiologists, geneticists, methodologists, statisticians, and journal editors to develop recommendations for the reporting of genetic risk prediction studies - the GRIPS statement.

## Genetic risk prediction studies

Genetic risk prediction studies typically develop or validate models that predict the risk of disease, but they are also being investigated for use in predicting prognostic outcome, treatment response, or treatment-related harms. Risk prediction models are statistical algorithms, which may be simple genetic risk scores (for example, risk allele counts), may be based on regression analyses (for example, weighted risk scores or predicted risks), or may be based on more complex analytic approaches, such as support vector machine learning or classification trees. The risk models may be based on genetic variants only, or include both genetic and non-genetic risk factors [14].

## Aims and use of the GRIPS statement

The 25 items of the GRIPS statement are intended to maximize the transparency, quality, and completeness of reporting on research methodology and findings in a particular study. It is important to emphasize that these recommendations are guidelines only for how to report research and do not prescribe how to perform genetic risk prediction studies. The guidelines do not support or oppose the choice of any particular study design or method; for example, the guidelines recommend that the study population should be described, but do not specify which population is preferred in a particular study.

The intended audience for the reporting guidelines is broad and includes epidemiologists, geneticists, statisticians, clinician scientists, and laboratory-based investigators who undertake genetic risk prediction studies, as well as journal editors and reviewers who have to appraise the design, conduct and analysis of such studies. In addition, it includes 'users' of such studies who wish to understand the basic premise, design, and limitations of genetic prediction studies in order to interpret the results for their potential application in health care. These guidelines are also intended to ensure that essential data from future genetic risk prediction studies are presented in standardized form, which will facilitate information synthesis as part of systematic reviews and meta-analyses.

Items presented in the checklist are relevant for a wide array of risk prediction studies, because GRIPS focuses on the main aspects of the design and analysis of risk prediction studies. GRIPS does not address randomized trials that may be performed to test risk models, nor does it specifically address decision analyses, cost-effectiveness analyses, assessment of health care needs, or assessment of barriers to health care implementation [15]. Once the performance of a risk model has been established, these next steps toward implementation require further evaluation [10,16]. For the reporting of these studies, which go beyond the assessment of genetic risk models as such, additional requirements apply. However, proper documentation of genetic predictive research according to GRIPS might facilitate the translation of research findings into clinical and public health practice.

## Development of the GRIPS statement

The GRIPS statement was developed by a multidisciplinary panel of 25 risk prediction researchers, epidemiologists, geneticists, methodologists, statisticians, and journal editors, seven of whom were also part of the STREGA initiative [11]. They attended a two-day meeting in Atlanta, Georgia (US) in December 2009 that was sponsored by the US Centers for Disease Control and Prevention on behalf of the Human Genome Epidemiology Network (HuGENet) [17]. Participants discussed a draft version of the guidelines that was prepared and distributed before the meeting. This draft version was developed on the basis of existing reporting guidelines, namely STREGA [11], REMARK [13], and STARD [12]. These were selected out of all available guidelines [18] because of their focus on observational study designs and genetic factors (STREGA), prediction models (REMARK), and test evaluation (REMARK and STARD). During the meeting, methodological issues pertinent to risk prediction studies were addressed in presentations. Workshop participants were asked to change, combine, or delete proposed items and add additional items if necessary. Participants had extensive post-meeting electronic correspondence. To harmonize our recommendations for genetic risk prediction studies with previous guidelines, we chose the same wording for the items wherever possible. Finally, we tried to create consistency with previous guidelines for the evaluation of risk prediction studies of cardiovascular diseases and cancer [2,19]. The final version of the checklist is presented in Table 1.

**Table 1:** Reporting recommendations for evaluation of risk prediction models that include genetic variants

**Title and abstract**	1	(a) Identify the article as a study of risk prediction using genetic factors. (b) Use recommended keywords in the abstract: genetic or genomic, risk, prediction
		
**Introduction**		
Background and rationale	2	Explain the scientific background and rationale for the prediction study
Objectives	3	Specify the study objectives and state the specific model(s) that is/are investigated. State if the study concerns the development of the model(s), a validation effort, or both
		
**Methods**		
Study design and setting	4*	Specify the key elements of the study design and describe the setting, locations, and relevant dates, including periods of recruitment, follow-up, and data collection
Participants	5*	Describe eligibility criteria for participants, and sources and methods of selection of participants
Variables: definition	6*	Clearly define all participant characteristics, risk factors and outcomes. Clearly define genetic variants using a widely used nomenclature system
Variables: assessment	7*	(a) Describe sources of data and details of methods of assessment (measurement) for each variable. (b) Give a detailed description of genotyping and other laboratory methods
Variables: coding	8	(a) Describe how genetic variants were handled in the analyses. (b) Explain how other quantitative variables were handled in the analyses. If applicable, describe which groupings were chosen, and why
Analysis: risk model construction	9	Specify the procedure and data used for the derivation of the risk model. Specify which candidate variables were initially examined or considered for inclusion in models. Include details of any variable selection procedures and other model-building issues. Specify the horizon of risk prediction (for example, 5-year risk)
Analysis: validation	10	Specify the procedure and data used for the validation of the risk model
Analysis: missing data	11	Specify how missing data were handled
Analysis: statistical methods	12	Specify all measures used for the evaluation of the risk model, including, but not limited to, measures of model fit and predictive ability
Analysis: other	13	Describe all subgroups, interactions, and exploratory analyses that were examined
		
**Results**		
Participants	14*	Report the numbers of individuals at each stage of the study. Give reasons for non-participation at each stage. Report the number of participants not genotyped, and reasons why they were not genotyped
Descriptives: population	15*	Report demographic and clinical characteristics of the study population, including risk factors used in the risk modeling
Descriptives: model estimates	16	Report unadjusted associations between the variables in the risk model(s) and the outcome. Report adjusted estimates and their precision from the full risk model(s) for each variable
Risk distributions	17*	Report distributions of predicted risks and/or risk scores
Assessment	18	Report measures of model fit and predictive ability, and any other performance measures, if pertinent
Validation	19	Report any validation of the risk model(s)
Other analyses	20	Present results of any subgroup, interaction, or exploratory analyses, whenever pertinent
		
**Discussion**		
Limitations	21	Discuss limitations and assumptions of the study, particularly those concerning study design, selection of participants, and measurements and analyses, and discuss their impact on the results of the study
Interpretation	22	Give an overall interpretation of results considering objectives, limitations, multiplicity of analyses, results from similar studies, and other relevant evidence
Generalizability	23	Discuss the generalizability and, if pertinent, the health care relevance of the study results
		
**Other**		
Supplementary information	24	State whether databases for the analyzed data, risk models, and/or protocols are or will become publicly available and, if so, how they can be accessed
Funding	25	Give the source of funding and the role of the funders for the present study. State whether there are any conflicts of interest

Items marked with an asterisk should be reported for every population in the study.

## The GRIPS explanation and elaboration article

Accompanying this GRIPS statement, an Explanation and Elaboration document has been written [20], modeled after those developed for other reporting guidelines [21-24]. The Explanation and Elaboration document illustrates each item with at least one published example that we consider transparent in reporting, explains the rationale for its inclusion in the checklist, and presents details of the items that need to be addressed to ensure transparent reporting. The Explanation and Elaboration document was produced after the meeting. The document was prepared by a small subgroup and shared with all workshop participants for additional revisions and final approval.

## Conclusions and future directions

High-quality reporting reveals the strengths and weaknesses of empirical studies, facilitates the interpretation of the scientific and health care relevance of the results - especially within the framework of systematic reviews and meta-analyses - and helps build a solid evidence base for moving genomic discoveries into applications in health care practice. The GRIPS guidelines were developed to improve the transparency, quality and completeness of the reporting of genetic risk prediction studies. As outlined in the introduction, GRIPS does not prescribe how studies should be designed, conducted, or analyzed, and therefore the guidelines should not be used to assess the quality of empirical studies [25]. The guidelines should be used only to check whether all essential items are adequately reported.

Finally, the methodology for designing and assessing genetic risk prediction models is still developing. For example, newer measures of reclassification were first introduced in 2007 [26], and several alternative reclassification measures have been proposed [27]. Which measures to apply and when to use measures of reclassification are still subject to ongoing evaluation and discussion [28]. Furthermore, alternative strategies for constructing risk models other than simple regression analyses are being explored, and these may add increased complexity to the reporting. In formulating the items of the GRIPS statement, these methodological advances were anticipated. It is for this reason that the GRIPS statement recommends how a study should be reported and not how a study should be conducted or analyzed. Therefore, methodological and analytical developments will not immediately impact the validity and relevance of the items, but the GRIPS statement will be updated when this is warranted by essential new developments in the construction and evaluation of genetic risk models.

## Co-publication

In order to encourage dissemination of the GRIPS statement, this article will also be published by *PLoS Medicine*, *Annals of Internal Medicine*, *BMJ*, *Circulation: Cardiovascular Genetics*, *European Journal of Clinical Investigation*, *European Journal of Epidemiology*, *European Journal of Human Genetics*, *Genetics in Medicine*, *Genome Medicine*, and *Journal of Clinical Epidemiology*.

## Abbreviations

GRIPS: Genetic RIsk Prediction Studies; REMARK: Reporting of tumor MARKer studies; STARD: STAndards for Reporting Diagnostic accuracy; STREGA: STrenghtening the REporting of Genetic Association studies.

## Competing interests

The authors declare that they have no competing interests.
